# Validation of the International Classification of Functioning, Disability and Health (ICF) Core Set for rheumatoid arthritis from the patient perspective using focus groups

**DOI:** 10.1186/ar1956

**Published:** 2006-05-09

**Authors:** Michaela Coenen, Alarcos Cieza, Tanja A Stamm, Edda Amann, Barbara Kollerits, Gerold Stucki

**Affiliations:** 1 ICF Research Branch of the WHO Collaborating Center for the Family of International Classifications at the German Institute of Medical Documentation and Information (DIMDI), IHRS, Marchioninistraße 17, 81377 Munich, Germany; 2 Department of Physical Medicine and Rehabilitation, University Hospital Munich, Marchioninistraße 15, 81377 Munich, Germany; 3 Vienna Medical University, Department of Internal Medicine III, Division of Rheumatology, Waehringer Guertel 18–20, 1090 Vienna, Austria; 4 Swiss Paraplegic Research (SPF), Nottwil, Switzerland

## Abstract

Functioning is recognized as an important study outcome in rheumatoid arthritis (RA). The Comprehensive ICF Core Set for RA is an application of the International Classification of Functioning, Disability and Health (ICF) of the World Health Organisation with the purpose of representing the typical spectrum of functioning of patients with RA. To strengthen the patient perspective, persons with RA were explicitly involved in the validation of the Comprehensive ICF Core Set for RA using qualitative methodology. The objective of the study was twofold: to come forward with a proposal for the most appropriate methodology to validate Comprehensive ICF Core Sets from the patient perspective; and to add evidence to the validation of the Comprehensive ICF Core Set for RA from the perspective of patients. The specific aims were to explore the aspects of functioning and health important to patients with RA using two different focus group approaches (open approach and ICF-based approach) and to examine to what extent these aspects are represented by the current version of the Comprehensive ICF Core Set for RA. The sampling of patients followed the maximum variation strategy. Sample size was determined by saturation. The focus groups were digitally recorded and transcribed verbatim. The meaning condensation procedure was used for the data analysis. After qualitative data analysis, the resulting concepts were linked to ICF categories according to established linking rules. Forty-nine patients participated in ten focus groups (five in each approach). Of the 76 ICF categories contained in the Comprehensive ICF Core Set for RA, 65 were reported by the patients based on the open approach and 71 based on the ICF-based approach. Sixty-six additional categories (open approach, 41; ICF-based approach, 57) that are not covered in the Comprehensive ICF Core Set for RA were raised. The existing version of the Comprehensive ICF Core Set for RA could be confirmed almost entirely by the two different focus group approaches applied. Focus groups are a highly useful qualitative method to validate the Comprehensive ICF Core Set for RA from the patient perspective. The ICF-based approach seems to be the most appropriate technique.

## Introduction

Functioning is recognized as an important study outcome in rheumatoid arthritis (RA). The number of clinical studies addressing functioning as a study endpoint in patients with RA has steadily increased during the past decade [1]. These investigations have predominantly been guided by the medical perspective, from which the measurement of functioning and health is required to evaluate the patient-relevant outcomes of an intervention and from which functioning and health are seen primarily as a consequence of the disease [2]. Many of these investigations include patient-oriented instruments, for example, patient and proxy self-reports on health status, quality of life, and health preferences. In rheumatology, the Health Assessment Questionnaire Disability Index (HAQ [3]) and the Arthritis Impact Measurement Scales (AIMS2 [4]), which can be considered a generic instrument specific for RA, are widely used.

These instruments have also been developed according to the medical perspective and in line with the current concept in outcomes and quality-of-life research of condition-specific measures [5], that is, they are based on the assumption that different conditions are associated with salient patient problems in functioning. The individual influence of the environment and personal factors is, however, rarely taken into account [6,7]. In addition, widely used RA-specific health-status measures, like the Health Assessment Questionnaire Disability Index, mainly address activities far more than participation [8]. However, patients' experiences of functioning are determined by their interaction with the environment and their own personal characteristics and not only by the health condition [9-12]. RA is also very much associated with the inability to continue working, ultimately leading to the experience of restriction in participation [13-16]. Thus, a very comprehensive approach is required when addressing RA.

**Figure 1:**
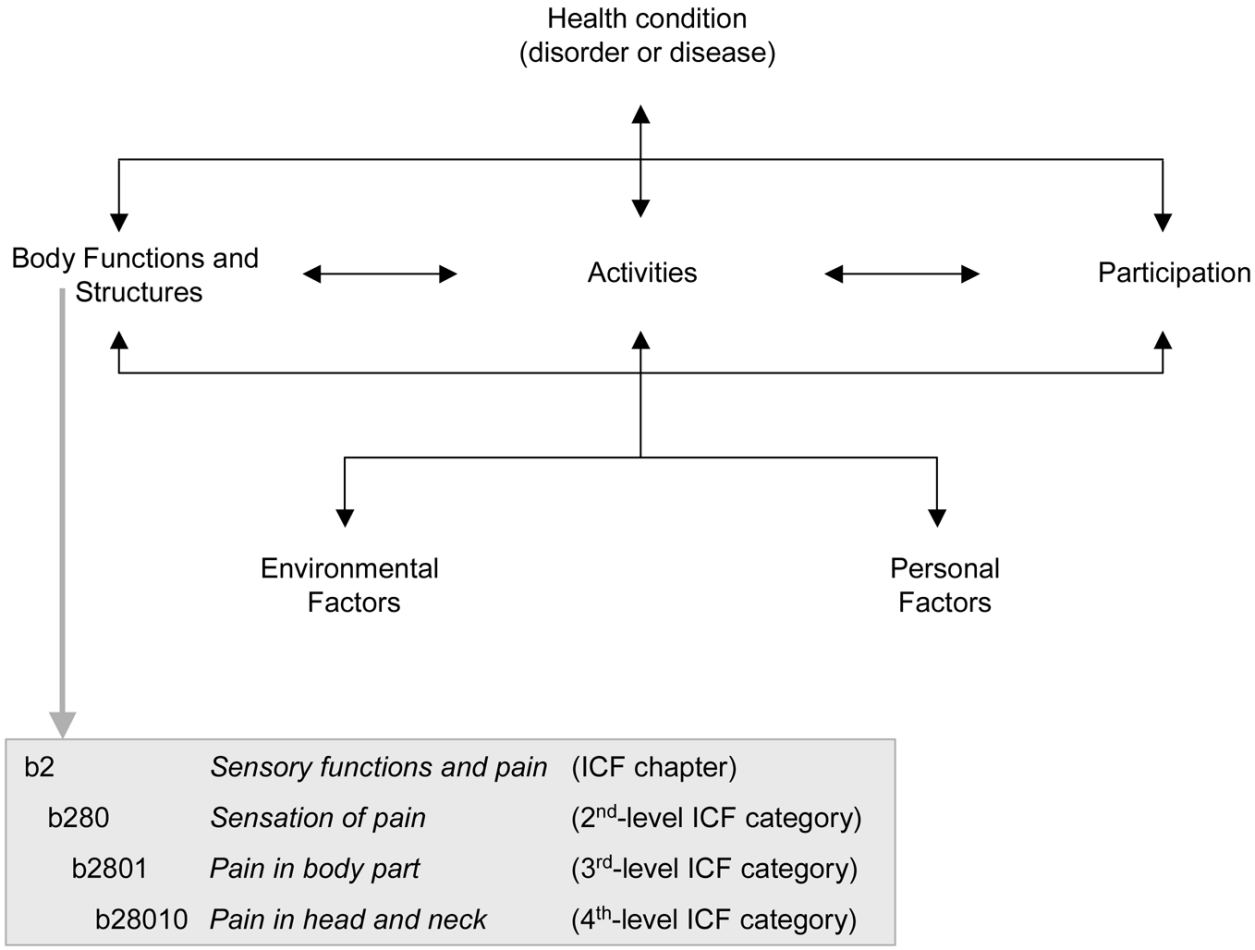
The bio-psycho-social model of functioning, disability and health.

The bio-psycho-social model of Functioning, Disability and Health of the World Health Organization (WHO) [17] establishes the basis for a more comprehensive description of the experience of patients suffering from determined disease. Based on this model, functioning, with its components 'Body Functions', 'Body Structures' and 'Activities and Participation', is seen in relation to the health condition under consideration, as well as 'Personal Factors' and 'Environmental Factors' (Figure 1) [17]. Functioning denotes the positive aspects, and disability the negative aspects of the interaction between an individual with a health condition and the contextual factors (Environmental Factors and Personal Factors) of that individual.

This bio-psycho-social view guided the development of the International Classification of Functioning, Disability and Health (ICF), which was approved by the World Health Assembly (WHA) in May 2001. As the ICF has been developed in a worldwide, comprehensive consensus process over the past few years and was endorsed by the WHA as a member of the WHO Family of International Classifications, it is likely to become the generally accepted framework to describe functioning and health. The ICF is intended for use in multiple sectors that, besides health, include education, insurance, labour, health and disability policy, statistics, and so on. In the clinical context, it is intended for use in needs assessment, matching interventions to specific health states, rehabilitation and outcome evaluation. With the ICF, not only an etiologically neutral framework, but a globally agreed-on language and a classification is available to describe functioning on both the individual and population levels and from both the patient perspective and that of the health professionals. The ICF contains more than 1,400 so-called ICF categories, each allotted to the named components in the bio-psycho-social model with the exception of the component Personal Factors, which has not yet been classified. Each ICF category is denoted by a code composed by a letter that refers to the components of the classification (b, Body Functions; s, Body Structures; d, Activities and Participation; e, Environmental Factors) and is followed by a numeric code starting with the chapter number (one digit), followed by the 2nd level (two digits) and the 3rd and 4th levels (one digit each) (Figure 1).

All member states of the WHO are now called upon to implement the ICF in multiple sectors that, besides health, include education, insurance, labour, health-and-disability policy, statistics, and so on. However, the ICF has to be tailored to suit these specific applications [18]. In the clinical context, the main challenge is the length of the classification with its over 1,400 categories. Mainly to address the issue of feasibility regarding the number of categories, ICF Core Sets have been developed in a formal decision making and consensus-based process integrating evidence gathered from preliminary studies for a number of the most burdensome, chronic health conditions, including RA [19]. The preliminary studies included a Delphi exercise [20], a systematic review [21] on outcomes used in randomized clinical trials, which represents the view of researchers performing studies, and an empirical data collection, using the ICF checklist [22]. Based on these studies, relevant ICF categories were identified. The lists of these identified categories represent the starting point of the decision-making and consensus process that took place at the consensus conference. The ICF Core Sets for patients with a determined health condition represent a selection of ICF categories out of the whole classification that can serve as minimal standards for the reporting of functioning and health for clinical studies and clinical encounters (Brief ICF Core Set) or as standards for multiprofessional, comprehensive assessment (Comprehensive ICF Core Set) under consideration of influential Environmental Factors. Since the ICF Core Sets address aspects within all the components of the ICF (Body Functions, Body Structures, Activities and Participation, Environmental Factors) they present a broad, condition-specific perspective that may reflect the whole health experience of patients. The current version of the Comprehensive ICF Core Set for RA includes 76 categories at the 2nd, 8 categories at the 3rd, and 12 categories at the 4th level of the classification. Regarding the 2nd level of the classification, 15 categories pertain to the component Body Functions, 8 categories to the component Body Structures, 32 categories to the component Activities and Participation and 21 categories to the component Environmental Factors [23]. The Comprehensive ICF Core Set for RA describes the typical spectrum of problems in functioning among patients with RA encountered in comprehensive assessments or in clinical studies. Additionally, it provides an ideal basis from which to define theoretically sound models of functioning and disability in patients with RA.

The Comprehensive ICF Core Set for RA is now undergoing worldwide testing and validation using a number of approaches, including an international multicenter validation study and validation from the perspective of health professionals. One key aspect is the validation from the patient perspective. While the patient perspective has been implicitly included in the development of ICF Core Sets [22], the patients now will be explicitly involved in the process of the development and validation of ICF Core Sets. As standards of functioning and health in research and clinical practice, the ICF Core Sets have to show that they address the perspective of those who experience the disease.

Qualitative methodology provides the possibility to explore the perspective of those who experience a health problem, that is, the so-called patient perspective [24,25]. Qualitative methods are now widely used and increasingly accepted in health research and health-related sciences [26-28]. One of the most broadly used techniques in qualitative research is the focus group methodology [29-31]. Focus groups are "carefully planned series of discussions designed to obtain perceptions on a defined area of interest in a permissive, non-threatening environment" [32]. They are especially useful for studies that involve complex issues that entail many levels of feeling and experience [33]. "The basic goal in conducting focus groups is to hear from the participants about the topics of interest to the researcher" [34]. The idea behind this methodology is that group processes can help people to explore and clarify their views [35]. The non-directive nature of focus groups affords participants an opportunity to comment, explain, disagree and share experiences and attitudes [36].

The objective of the present study was twofold: first, to come forward with a proposal for the most appropriate focus group approach to validate Comprehensive ICF Core Sets from the patient perspective; and second, to add evidence to the validation of the Comprehensive ICF Core Set for RA from the perspective of patients with RA based on a group of German patients. The specific aims were to explore the aspects of functioning and health important to patients with RA using two different focus group approaches and to examine to what extent these aspects are represented by the current version of the Comprehensive ICF Core Set for RA.

## Materials and methods

### Design

We conducted a qualitative study with patients with RA using the focus group methodology. Two different focus group approaches were used, an open approach and an ICF-based approach. In the open approach, open-ended questions asking the patients to name their problems in Body Functions, Body Structures, and Activities and Participation were used. The patients were additionally asked about Environmental Factors (barriers and facilitators) influencing their everyday life (Table 1). In the ICF-based approach, each of the titles of the ICF chapters from which categories are included in the Comprehensive ICF Core Set for RA were presented. For each of the presented chapters, open-ended questions on possible problems in each of the life areas that the ICF chapters represent were used (Table 1). Finally, the patients were asked whether they thought anything was missing in the Comprehensive ICF Core Set for RA.

The study was approved by the Ethics Commission of the Ludwig-Maximilian University, Munich.

### Participants

All patients with RA diagnosed according to the revised American College of Rheumatology Criteria [37] who had been treated in the day clinic of the Department of Physical Medicine and Rehabilitation of the Ludwig-Maximilian University in Munich at any time since 2001 were contacted by mail and asked whether they would like to participate in the study. Participants were then selected from the list of all willing patients by the maximum variation strategy [38] based on the criteria disease duration and age group. Further participants were recruited from the German self-help service ('Deutsche Rheuma-Liga e.V.'). The group size was set at a maximum of seven persons to represent different opinions and facilitate interactions. Patients who participated in the focus groups gave written informed consent according to the Declaration of Helsinki 1996.

### Sample size

The sample size was determined by saturation [38]. Saturation refers to the point at which an investigator has obtained sufficient information from the field [32] (see Data analysis: saturation of data).

### Data collection

All groups were conducted in a non-directive manner by the same moderator (MC) and one group assistant (EA, 'open approach'; BK, 'ICF-based approach'). Moderator and group assistants were psychologists with expertise in the ICF and in conducting group processes.

The focus groups were conducted according to focus group guidelines, including open-ended questions and further instructions (for example, introduction, procedure of the session, technical aspects). At the beginning of each focus group, the procedure of the session was explained, and the concept of the ICF was presented in lay terms to all participants. Then one of the two different focus group approaches was performed (open approach or ICF-based approach). The open-ended questions or the titles of the chapters (ICF-based approach) were presented visually to the participants by a Microsoft PowerPoint presentation. At the end of each focus group, a summary of the main results was given back to the group to enable the participants to verify and amend emergent issues.

The focus groups were digitally recorded and transcribed verbatim with an Olympus DSS system. The assistants observed the process within the group. Additionally, they filled in field notes according to a standardized coding schema. Field notes refer descriptive observations of the group interaction and of the topics of discussion. After each focus group a debriefing with moderator and assistant took place to review the course of the focus group.

The two focus group approaches were conducted alternately.

### Data analysis

#### Qualitative analysis

The meaning condensation procedure [39] was used for the qualitative analysis of data. In the first step, the transcripts of the focus groups were read through to get an overview over the collected data. In the second step, the data were divided into units of meaning, and the theme that dominated a meaning unit was determined. A meaning unit was defined as a specific unit of text either a few words or a few sentences with a common theme [40]. Therefore, a meaning unit division did not follow linguistic grammatical rules. Rather, the text was divided where the researcher discerned a shift in meaning [39]. In the third step, the concepts contained in the meaning units were identified. A meaning unit could contain more than one concept.

#### Linking to the ICF

According to the purpose of multiple coding, the identified concepts were linked to the categories of the ICF by two health professionals (open approach, MC and EA; ICF-based approach, MC and BK) based on established linking rules [6,7], which enable linking concepts to ICF categories in a systematic and standardized way (Table 2). According to these linking rules, health professionals trained in the ICF are advised to link each concept to the ICF category representing this concept most precisely. One concept could be linked to one or more ICF categories, depending on the number of themes contained in the concept. Consensus between the two health professionals was used to decide which ICF category should be linked to each identified concept. In case of a disagreement, a third person trained in the linking rules was consulted. In a discussion led by the third person, the two health professionals that linked the concepts stated their pros and cons for the linking of the concept under question to a specific ICF category. Based on these statements, the third person made an informed decision.

#### Saturation of data

In this study saturation was defined as the point during data collection and analysis when the linking of the concepts of two consecutive focus groups reveals no additional 2nd level categories of the Comprehensive ICF Core Set for RA with respect to previous focus groups. Saturation was checked separately for the two approaches.

#### Confirmation of the ICF categories contained in the Comprehensive ICF Core Set for RA

An ICF category of the Comprehensive ICF Core Set for RA was regarded as confirmed if the identical or a similar category emerged from the focus groups (for example, s299 eye, ear and related structures, unspecified confirmed by s230 structures around eye). Since the ICF categories are arranged in a hierarchical code system, the 2nd level categories of the Comprehensive ICF Core Set for RA were considered confirmed when the corresponding 3rd or 4th level category of which they were a member had been named by the patients.

#### Accuracy of the analysis

To audit the accuracy of the analysis, 15% of the transcribed text was randomly selected, analyzed according to the meaning condensation procedure, and linked to the ICF by two health professionals (MC and TS) as a peer review. This process was performed in addition to the process described in the section 'Linking to the ICF'. The degree of agreement between the two investigators regarding the identified and linked concepts in this random selected text was calculated by kappa statistic with 95%-bootstrapped confidence intervals [41,42]. The values of the kappa coefficient generally range from 0 to 1, where 1 indicates perfect agreement and 0 indicates no additional agreement beyond what is expected by chance alone. The data analysis was performed with SAS for windows V9.1 (SAS Institute Inc., Cary, NC, USA).

## Results

### Description of the focus groups

**Figure 2:**
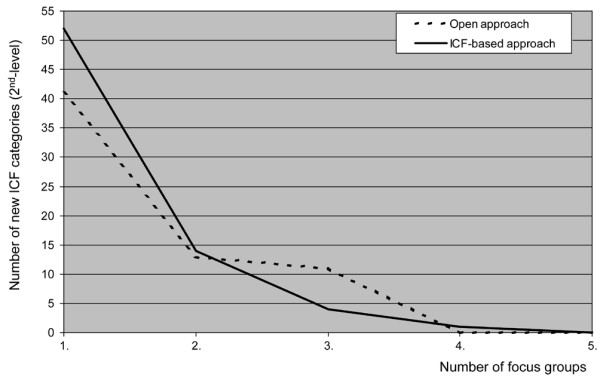
Saturation of the qualitative data in the focus groups. ICF, International Classification of Functioning, Disability and Health.

A total of 49 participants were included in the focus groups (open approach, *n *= 25; ICF-based approach, *n *= 24). Participants' characteristics are summarized in Table 3. Ten focus groups with five groups in each approach were conducted. The focus group sessions lasted from about fifty minutes to two hours, including a short break. Regarding the categories of the Comprehensive ICF Core Set for RA, saturation of data was reached in both approaches after conducting five focus groups (Figure 2).

### Qualitative analysis and linking

A total of 1,900 relevant concepts were identified in the two approaches (open approach, *n *= 897; ICF-based approach, *n *= 1,003). These concepts were linked to 342 different ICF categories. For 155 of the 342 categories at the 3rd and 4th level of the classification, the corresponding 2nd level categories were considered (*n *= 66). Thus, the concepts were linked to a total of 253 2nd level categories. Fifty-two concepts named by the participants were more specific than the corresponding most specific ICF category (for example, jaw joint, problems with climbing upstairs). Regarding the categories of the chapter 'sensory functions and pain' (b2), for example, the participants reported several issues according to the pain quality (pressure pain, rest pain, stabbing pain), which are not specifically covered by the existing ICF categories. Therefore, all these concepts referring to different qualities of pain were linked to the ICF category 'b280 sensation of pain'.

Thirty-two concepts could not be linked to ICF categories (for example, quality of life in general, aspects of coping, disease management, time-related aspects, and variability of functioning). Fifteen of them could be allotted to the component Personal Factors, which has not yet been classified.

### Confirmation of the Comprehensive ICF Core Set for RA

In total, 74 out of the 76 2nd level categories included in the Comprehensive ICF Core Set for RA were confirmed by the two focus group approaches (open approach, *n *= 65; ICF-based approach, *n *= 71). All 2nd level categories of the components Body Functions (*n *= 15) and Body Structures (*n *= 8) that are included in the Comprehensive ICF Core Set for RA were reported by the patients in the ICF-based focus group approach (Tables 4 to 7; categories in bold typeface).

### Additional categories

Sixty-six 2nd level additional categories (open approach, *n *= 41; ICF-based approach, *n *= 57) that are not included in the current version of the Comprehensive ICF Core Set for RA were identified in the focus groups (Tables 4 to 7). Most of the additional categories derive from the component Body Functions (open approach, *n *= 19; ICF-based approach, *n *= 29) followed by Environmental Factors (open approach, *n *= 15; ICF-based approach, *n *= 16). Five additional categories in the open approach and eight additional categories in the ICF-based approach were reported by the participants as related to the component Activities and Participation. Two and four additional categories from the component Body Structures were reported in the open and the ICF-based approach, respectively.

### Accuracy of the analysis

The kappa coefficient for the agreement between the two investigators (peer review) was 0.66. The 95%-bootstrapped confidence interval, which indicates the precision of the estimated kappa coefficient, was 0.61 to 0.73.

## Discussion

The current version of the Comprehensive ICF Core Set for RA could be confirmed almost entirely from the patient perspective by the two different focus group approaches applied (open approach and ICF-based approach). This study also confirmed relevant outcomes of treatment in RA from the patient perspective (for example, pain, stiffness, fatigue, mobility, muscle strength, getting social support) [24,43,44]. However, some issues emerged from the patient perspective that have not yet been covered by the Comprehensive ICF Core Set for RA or even by the ICF classification. ICF categories of the Comprehensive ICF Core Set for RA not reported by the patients were 'd570 looking after one's health' and 'e360 other professionals (support and relationship)'.

Sixty-six additional 2nd level categories that are not covered in the current version of the Comprehensive ICF Core Set for RA were raised. Most of the additional categories belong to the component Body Functions followed by the component Environmental Factors. Some of these additional ICF categories need special discussion.

It is important to emphasize that several categories were named by the patients at a higher level of specification than the 2nd level of the ICF. Some of these more specific categories are included in the Comprehensive ICF Core Set for RA, and some are not [23]. One of these very specific categories not included in the Comprehensive ICF Core Set for RA at higher levels of specification are 'fatigue' and 'fatiguability'. 'Fatigue' and 'fatiguability' were linked to the 3rd level category 'b1300 energy level' and 'b4552 fatiguability', which belong to the 2nd level categories 'b130 energy and drive functions' and 'b455 exercise tolerance functions', respectively. Fatigue was also identified as an area of particular importance to patients with RA at OMERACT (Outcome Measures in Rheumatoid Arthritis Clinical Trials) VI [25,45], as patient-relevant outcome in RA [44,46] and as an adverse effects of medication [47,48]. Our study might thus suggest that the categories 'b1300 energy level' and 'b4552 fatiguability' should be specifically and explicitly included in the Comprehensive ICF Core Set for RA. This suggestion is strengthened by the findings of an ICF Core Set validation study deriving individual interviews [49] and validation studies from the health-professionals perspective.

Numerous additional categories were related to side effects of medication, which are an important issue for satisfaction with treatment from the patient perspective [24,43,50]. The participants of the study explicitly attributed some categories from the components Body Functions and Body Structures to side effects. Some of these causal relationships can also be found in the literature as complications due to medication [51-59] or as relevant problems from the patient perspective [60,61]. The question whether ICF categories concerning side effects of medication should be included in the Comprehensive ICF Core Set for RA has to be considered carefully. With the advent of new medications, new side effects may appear. On one hand, one has to keep in mind that the ICF Core Sets establish the standards of 'what to measure' in patients with RA independent of the treatment (one could even say independent of 'fashionable treatment'). On the other hand, the intake of medication and the suffering of side effects belong to the reality of patients with RA. Perhaps one solution to this dilemma could be the development of treatment-specific ICF Core Sets.

Within the component Environmental Factors numerous categories not included in the current version of the Comprehensive ICF Core Set for RA were reported by the patients. There is no doubt that social support is an important Environmental Factor for patients with RA [62]. Several studies pointed out the relationship and interaction between social support and disease activity, pain or disability [63-65].

The category 'e165 (financial) assets', which is not included in the current version of the Comprehensive ICF Core Set for RA, was reported by the participants in the focus groups and in the ICF Core Set validation study using individual interviews [49] as a relevant Environmental Factor. Economic consequences in relation to income reduction or to loss of paid work due to physical disability were also found to be an important issue to patients with RA in the literature [63,66-68]. Within this context, it has to be taken into account that patients with RA in most countries also have substantial RA-related out-of-pocket medical expenditures for co-payments for prescribed drugs, over-the-counter drugs and costs to complementary and alternative medicine [69,70].

With both approaches used in this study, we found a broad range of themes that could be linked to the corresponding categories. Both approaches performed satisfactory results; however, it is important to mention that some patient-sensitive issues were only reported in the ICF-based approach, for example, 'b535 sensations associated with the digestive system', 'b610 urinary excretory functions', 'b620 urination functions', 'b640 sexual functions', and 'd530 toileting'. Issues concerning mood, disease management and coping were reported in detail in the open approach. Comparing the two approaches, the ICF-based approach seems to be the appropriate technique to confirm the Comprehensive ICF Core Set for RA, particularly with regard to the coverage of the components Body Structures and Body Functions.

In qualitative research and studies with focus group methodology, sample sizes typically remain small because intensive data analysis is required [30,32]. A small sample size with a diverse range of participants (*n *= 49) was used to obtain the required level of rich and meaningful data. According to Curtis and colleagues [71], the small samples in qualitative research are studied intensively and typically generate a large amount of information. By keeping the questions open-ended, the moderator can stimulate useful trains of thought in the participants that were not anticipated [72]. The focus groups in our study were composed of four to seven participants. We decided to include groups with few participants because of the complexity of the topic and the expertise of the participants according to the literature [73]. With a small group size, each participant has a greater opportunity to talk, which is reported as an important aspect for the group dynamics in groups with elderly and ill participants [30,74].

The characteristics of the sample in this study (gender, age, disease duration) are comparable to samples in other national [62,75] and international studies [43,63]. It is important to mention that several strategies were used to improve and verify the trustworthiness of the qualitative data. Triangulation was used to ensure the comprehensiveness of data. We included different aspects of triangulation by using two approaches to focus groups (methodological triangulation) and two data analysts (investigator triangulation: multiple coding) [76,77]. Continuous data analysis was used according to Pope and colleagues [78]. Reflexivity was assured by conducting a research diary for the documentation of memos concerning the design, data collection and analysis of the study. Clear exposition was used to establish guidelines for conducting the focus groups (including open-ended questions), verbatim transcription, and linking rules [6,7]. Thus, a clear account of methods of data collection and analysis was assured. Finally, peer review was used. The kappa coefficient of 0.66 (0.61 to 0.73) for the accuracy of the peer review is comparable to other studies reporting kappa statistics about the linking of categories [49] and can be regarded as 'substantial agreement' [79].

There are also some limitations of this study that need special mention. The sample consists only of German participants. We conducted this study as a pilot study to develop an appropriate method to validate ICF Core Sets from the patient perspective. Our suggestion is that our methods could be used in similar studies in other countries to establish a cross-cultural perspective. Secondly, the linking process was performed by two health professionals according to established linking rules [6,7]. However, it remains unclear whether other health professionals would have decided differently. Finally, we followed the strategy of saturation during data analyses with the criteria of two consecutive focus groups revealing no additional 2nd level categories in the Comprehensive ICF Core Set for RA with respect to previous focus groups. Participants in a sixth focus group still might report new themes and concepts not yet reported.

Further research in the context of the development and confirmation of ICF Core Sets is needed. The results of this study will be presented at an international WHO conference and will be taken into account for the decision on the final version of the Comprehensive ICF Core Set for RA. In addition, using this study as a starting point, we are currently conducting further focus group studies with RA patients in other countries to implement the international perspective of the ICF Core Sets. Finally, the results of this study have also influenced the protocol that establishes the methods to developing ICF Core Sets. From now on the collection of data from the patient perspective will be implemented in the preliminary phase of the development of ICF Core Sets [19].

## Conclusion

It is extremely important to consider the patient perspective for the validation of the Comprehensive ICF Core Set for RA. The existing version of the Comprehensive ICF Core Set for RA with its selected categories could be confirmed almost entirely by the two different focus group approaches applied. Focus groups are a highly useful qualitative method to validate the Comprehensive ICF Core Set for RA from the patient perspective. The ICF-based approach, which uses the contents of the ICF Core Sets to structure the focus groups seems to be the most appropriate technique. Additional categories not represented in the Comprehensive ICF Core Set for RA emerged from the focus groups.

## Abbreviations

ICF = International Classification of Functioning, Disability and Health; RA = rheumatoid arthritis; WHA = World Health Assembly; WHO = World Health Organization.

## Competing interests

The authors declare that they have no competing interests.

## Authors' contributions

MC conceived and organized the study and drafted the manuscript. TS participated in the development of the focus group guidelines, the drafting of the manuscript and was involved in the peer review. AC participated in the development of the study design and accompanied the study implementation. EA and BK shared the focus groups as assistants and undertook the task of linking the qualitative data to the ICF. GS was responsible for the overall design of the development and the validation of ICF Core Sets.

## Figures and Tables

**Table 1:** Open-ended questions of the focus groups

Open-ended questions

If you think about your body and mind, what does not work the way it is supposed to?
If you think about your body, in which parts are your problems?
If you think about your daily life, what are your problems?
If you think about your environment and your living conditions,:
what do you find helpful or supportive?
what barriers do you experience?

**Table 2:** Scheme of the qualitative data analysis

Transcription		Meaning unit	ICF category
Moderator:	*If you think about your body, what functional problems do you have?*		
Patient A:	I used to go to **sports **very often. Now I can't anymore. I even had to **quit swimming**	Restriction of sports Quit swimming	d9201 sports d4554 swimming
Patient B:	Exactly! I also had to **quit swimming**.		
Patient C:	**I can no longer cycle. **(...)	Quit cycling	d4750 driving human-powered transportation
Moderator:	*If you think about your body, where are your biggest problems?*		
Patient C:	**Toes, ankle joints, knee joints, fingers**	Toes Ankle joints	s7502 structure of ankle & foot s75021 ankle joint & joints of foot and toes
		Knee joints	s75011 knee joint
		Fingers	s7302 structure of hand
Patient A:	What bothers me are my **wrists. **(...)	Wrists	s73021 joints of hand and fingers

The transcription undergoes qualitative analysis to derive a meaning unit that is then linked to an International Classification of Functioning, Disability and Health (ICF) category.

**Table 3:** Characteristics of participants and focus groups

Characteristics of participants and focus groups	Focus groups		Total
	Open approach	ICF-based approach	
Number of participants (*n*)	25	24	49
Mean age, year (range)	59 (24–81)	54 (35–75)	57 (24–81)
Gender (% female)	88	83	86
Mean disease duration, year (range)	16 (4–38)	15 (3–56)	15 (3–56)
Number of focus groups (*n*)	5	5	10
Mean session duration, hours:minutes (range)	1:09 (0:52–1:15)	1:35 (1:17–2:06)	1:22 (0:52–2:06)

ICF, International Classification of Functioning, Disability and Health.

**Table 4:** Body Functions (b): patients' reporting of ICF categories (2^nd ^level)

ICF categories		Reported by patients	
Code	Title	Open approach	ICF-based approach

b126	Temperament and personality functions	Yes	Yes
**b130**	**Energy and drive functions**	**Yes**	**Yes**
**b134**	**Sleep functions**	**Yes**	**Yes**
b140	Attention functions	Yes	Yes
b147	Psychomotor functions	Yes	Yes
**b152**	**Emotional functions**	Yes	Yes
b160	Thought functions	Yes	Yes
**b180**	**Experience of self and time functions**		Yes
b210	Seeing functions	Yes	Yes
b215	Functions of structures adjoining the eye	Yes	
b220	Sensations associated with the eye and adjoining structures	Yes	Yes
b230	Hearing functions	Yes	Yes
b240	Sensations associated with hearing and vestibular function		Yes
b250	Taste function		Yes
b255	Smell function		Yes
b265	Touch function	Yes	Yes
b270	Sensory functions related to temperature and other stimuli		Yes
**b280**	**Sensation of pain**	**Yes**	**Yes**
b320	Articulation functions	Yes	
b410	Heart functions		Yes
**b430**	**Haematological system functions**	Yes	Yes
b435	Immunological system functions	Yes	Yes
**b455**	**Exercise tolerance functions**	**Yes**	**Yes**
b460	Sensations associated with cardiovascular and respiratory functions		Yes
**b510**	**Ingestion functions**	Yes	Yes
b515	Digestive functions	Yes	Yes
b520	Assimilation functions	Yes	
b525	Defecation functions	Yes	Yes
b530	Weight maintenance functions	Yes	Yes
b535	Sensations associated with the digestive system		Yes
b540	General metabolic functions		Yes
b545	Water, mineral and electrolyte balance functions	Yes	
b610	Urinary excretory functions		Yes
b620	Urination functions		Yes
**b640**	**Sexual functions**		Yes
**b710**	**Mobility of joint functions**	**Yes**	**Yes**
**b715**	**Stability of joint functions**	Yes	Yes
**b730**	**Muscle power functions**	**Yes**	**Yes**
**b740**	**Muscle endurance functions**		**Yes**
b760	Control of voluntary movement functions	Yes	Yes
**b770**	**Gait pattern functions**	**Yes**	**Yes**
**b780**	**Sensations related to muscles and movement functions**	**Yes**	**Yes**
b810	Protective functions of the skin	Yes	Yes
b820	Repair functions of the skin		Yes
b840	Sensation related to the skin		Yes
b850	Functions of hair		Yes
b860	Functions of nails		Yes

International Classification of Functioning, Disability and Health (ICF) categories of the ICF Core Set for rheumatoid arthritis are shown in bold typeface.

**Table 5:** Body Structures (s): patients' reporting of ICF categories (2^nd ^level)

ICF categories		Reported by patients	
Code	Title	Open approach	ICF-based approach

**s299**	**Eye, ear and related structures, unspecified **(s220)^a^	**Yes**	**Yes**
s320	Structure of mouth		Yes
s410	Structure of cardiovascular system	Yes	
s530	Structure of stomach	Yes	Yes
s540	Structure of intestine		Yes
**s710**	**Structure of head and neck region**	**Yes**	**Yes**
**s720 **	**Structure of shoulder region**	**Yes**	**Yes**
**s730**	**Structure of upper extremity**	**Yes**	**Yes**
**s750 **	**Structure of lower extremity**	**Yes**	**Yes**
**s760**	**Structure of trunk**	**Yes**	**Yes**
**s770**	**Additional musculoskeletal structures related to movement**	**Yes**	**Yes**
**s810**	**Structure of areas of skin**	**Yes**	**Yes**
s830	Structure of nails		Yes

International Classification of Functioning, Disability and Health (ICF) categories of the ICF Core Set for rheumatoid arthritis are shown in bold typeface.

**Table 6:** *Activities and Participation *(d): Patients' reporting of ICF categories (2nd level)

ICF categories		Reported by patients	
Code	Title	Open approach	ICF-based approach

d163	Thinking		Yes
d166	Reading		Yes
**d170**	**Writing**	**Yes**	**Yes**
d210	Undertaking a single task		Yes
**d230**	**Carrying out daily routine**	**Yes**	**Yes**
d240	Handling stress and other psychological demands		Yes
d330	Speaking	Yes	
**d360**	**Using communication devices and techniques**	**Yes**	**Yes**
**d410**	**Changing basic body position**	**Yes**	**Yes**
**d415**	**Maintaining a body position**	**Yes**	**Yes**
**d430**	**Lifting and carrying objects**	**Yes**	**Yes**
d435	Moving objects with lower extremities	Yes	
**d440**	**Fine hand use**	**Yes**	**Yes**
**d445**	**Hand and arm use**	**Yes**	**Yes**
**d449**	**Carrying, moving and handling objects, other specified and unspecified **(d430/d445)^a^	**Yes**	**Yes**
**d450**	**Walking**	**Yes**	**Yes**
**d455**	**Moving around**	**Yes**	**Yes**
**d460**	**Moving around in different locations **(d455)^a^	**Yes**	**Yes**
**d465**	**Moving around using equipment**	**Yes**	**Yes**
**d470**	**Using transportation**	**Yes**	**Yes**
**d475**	**Driving**	**Yes**	**Yes**
**d510**	**Washing oneself**	**Yes**	**Yes**
**d520**	**Caring for body parts**	**Yes**	**Yes**
**d530**	**Toileting**		**Yes**
**d540**	**Dressing**	**Yes**	**Yes**
**d550**	**Eating**		**Yes**
**d560**	**Drinking**	**Yes**	
**d570**	**Looking after one's health**	**No**	**No**
d610	Acquiring a place to live		Yes
**d620**	**Acquisition of goods and services **	**Yes**	**Yes**
**d630**	**Preparing meals**	**Yes**	**Yes**
**d640**	**Doing housework**	**Yes**	**Yes**
d650	Caring for household objects	Yes	Yes
**d660**	**Assisting others**		**Yes**
d720	Complex interpersonal interactions	Yes	Yes
d750	Informal social relationships	Yes	Yes
**d760**	**Family relationships**		**Yes**
**d770**	**Intimate relationships**	**Yes**	**Yes**
**d850**	**Remunerative employment**	**Yes**	**Yes**
**d859**	**Work and employment, other specified and unspecified **(d850)^a^	**Yes**	**Yes**
**d910**	**Community life**		**Yes**
**d920 **	**Recreation and leisure**	**Yes**	**Yes**

International Classification of Functioning, Disability and Health (ICF) categories of the ICF Core Set for rheumatoid arthritis are shown in bold typeface. ^a^Confirmation according to similar categories

**Table 7:** Environmental Factors (e): patients' reporting of ICF categories (2nd level)

ICF categories		Reported by patients	
Code	Title	Open approach	ICF-based approach

**e110**	**Products or substances for personal consumption**	**Yes**	**Yes**
**e115**	**Products and technology for personal use in daily living**	**Yes**	**Yes**
**e120**	**Products and technology for personal indoor and outdoor mobility and transportation **	**Yes**	**Yes**
**e125**	**Products and technology for communication**	**Yes**	**Yes**
e130	Products and technology for education		Yes
**e135**	**Products and technology for employment**	**Yes**	
**e150**	**Design, construction and building products and technology of buildings for public use**	**Yes**	**Yes**
**e155**	**Design, construction and building products and technology of buildings for private use**	**Yes**	**Yes**
e160	Products and technology of land development	Yes	Yes
e165	Assets	Yes	Yes
e210	Physical geography		Yes
**e225**	**Climate**	**Yes**	**Yes**
**e310**	**Immediate family**	**Yes**	**Yes**
e315	Extended family	Yes	Yes
**e320**	**Friends**	**Yes**	**Yes**
e325	Acquaintances, peers, colleagues, neighbours and community members	Yes	Yes
e330	People in positions of authority	Yes	
**e340**	**Personal care providers and personal assistants**	**Yes**	**Yes**
e345	Strangers	Yes	Yes
e350	Domesticated animals	Yes	Yes
**e355**	**Health professionals**	**Yes**	**Yes**
**e360**	**Other professionals**	**No**	**No**
**e410**	**Individual attitudes of immediate family members**	**Yes**	**Yes**
**e420**	**Individual attitudes of friends**	**Yes**	**Yes**
**e425**	**Individual attitudes of acquaintances, peers, colleagues, neighbors and community members**	**Yes**	**Yes**
e430	Individual attitudes of people in positions of authority		Yes
e445	Individual attitudes of strangers	Yes	Yes
**e450**	**Individual attitudes of health professionals**		**Yes**
**e460**	**Societal attitudes**	**Yes**	**Yes**
e465	Social norms, practices and ideologies	Yes	
e510	Services, systems and policies for the production of consumer goods	Yes	Yes
e515	Architecture and construction services, systems and policies	Yes	
**e540**	**Transportation services, systems and policies**	**Yes**	
e555	Associations and organizational services, systems and policies	Yes	Yes
e560	Media services, systems and policies	Yes	Yes
e565	Economic services, systems and policies		Yes
**e570**	**Social security services, systems and policies**	**Yes**	**Yes**
e575	General social support services, systems and policies	Yes	Yes
**e580**	**Health services, systems and policies**	**Yes**	**Yes**
e590	Labor and employment services, systems and policies	Yes	Yes

International Classification of Functioning, Disability and Health (ICF) categories of the ICF Core Set for rheumatoid arthritis are shown in bold typeface.
